# Multimedia-based hormone therapy information program for patients with prostate cancer: the result of a randomized pilot study

**DOI:** 10.1038/s41598-023-50006-6

**Published:** 2023-12-27

**Authors:** Ching-Hui Chien, Kuan-Lin Liu, Cheng-Keng Chuang, Chun-Te Wu, Ying-Hsu Chang, Kai-Jie Yu

**Affiliations:** 1 School of Nursing, National Taipei University of Nursing and Health Sciences, No.365, Ming-te Road, Peitou District, Taipei City, 112 Taiwan; 2 Division of Urology, Department of Surgery, Chang Gung Memorial Hospital at Keelung, Keelung City, Taiwan; 3 Division of Urology, Department of Surgery, Chang Gung Memorial Hospital at Linkou, Tao-Yuan City, Taiwan; 4 College of Medicine, Chang Gung University, Tao-Yuan City, Taiwan; 5 Division of Urology, Department of Surgery, New Taipei City Municipal TuCheng Hospital, New Taipei City, Taiwan

**Keywords:** Psychology, Health care, Urology

## Abstract

Few studies have explored the feasibility and efficacy of a multimedia information intervention for patients with prostate cancer who are undergoing hormone therapy. Thus, the purpose of the study was to assess the feasibility, acceptability, and the preliminary results of a multimedia-based hormone therapy information program (HTIP) on positive thinking and quality of life (QOL; primary outcomes) as well as social support and self-efficacy (secondary outcomes) of patients with prostate cancer. Patients with prostate cancer who were receiving hormone therapy were recruited from hospitals. After completing the pre-test questionnaire, patients were randomly divided into the multimedia information group (MIG; *n* = 40) and the control group (CG; *n* = 40). Patients in the MIG received a multimedia-based HTIP once a week for 6 weeks. Data were collected at 8 and 12 weeks after the pre-test. Measurement variables included positive thinking, QOL, social support, self-efficacy, and satisfaction with the program. The recruitment rate and retention rate were calculated for assessment of feasibility. The study had a 96.3% retention rate, and patients in the MIG were satisfied with the program. Preliminary results showed that, compared with those in the CG, patients in the MIG tended to exhibit higher positive thinking, prostate cancer-specific QOL, and social support at 8 weeks and 12 weeks after pre-test; however, the effect did not reach a statistically significant level. A multimedia-based HTIP is considered feasible and acceptable in patients with prostate cancer who underwent hormone therapy. Further research with a larger sample size, patients with high homogeneity in early-stage disease and long-term follow-up is needed to assess the efficacy of the intervention program.

Trial registration: ClinicalTrials.gov (NCT04693910); Registered 05/01/2021.

## Introduction

In 2020, approximately 1.4 million men worldwide were diagnosed with prostate cancer, and approximately 0.36 million patients died as a consequence of this disease that year^1^. Hormone therapy has been used for the control of metastatic or high-risk prostate cancer and alleviation of its symptoms^2^. After patients receive the initial hormone therapy for two to three years, however, androgen-dependent prostate cancer may progress to androgen-independent prostate cancer, which requires further adjustment of the treatment program^3^. Patients with hormone therapy may experience the effects of the cancer and subsequent physical and psychological changes following treatment, including an increase in fat, loss of muscle tissue, cognitive decline, cardiovascular disease, erectile dysfunction, loss of libido, fatigue, and depression^2,4,5^. These changes may further affect the self-efficacy^6^, positive thinking^7^, and quality of life (QOL)^8,9^ of patients. Qualitative research has shown that patients experience depression, anger, and self-blame after being diagnosed with prostate cancer and undergoing hormone therapy. In addition, they become passive in their interpersonal interactions and are unable to enjoy leisure activities^5^.

The concept of quality of life is a subjective experience that is influenced by personal environment and health. It encompasses various aspects of well-being in physical, psychological, and social dimensions^10^. Within the realm of care and medical management, one objective is to enhance the quality of life for individuals dealing with prostate cancer^11–16^. Positive thinking pertains to an individual’s level of personal satisfaction and their pursuit of goals^17–20^. Research has indicated that prostate cancer patients with greater self-efficacy and social support tend to exhibit higher levels of positive thinking^21^. Furthermore, higher levels of positive thinking, coupled with increased self-efficacy and improved social support, tend to result in an enhanced overall quality of life for these individuals^17,22^. Therefore, learning to prevent, care for, and respond to possible physical and psychosocial changes is crucial for patients with prostate cancer who undergo hormone therapy. Nevertheless, patients feel that they do not receive sufficient information support in the self-care process^5^.

### Efficacy of a multimedia-based information program on patients with prostate cancer

An information and education program can provide patients with disease-related knowledge, coping strategies, and stress management skills, which can be considered a type of psychosocial intervention^23^. Compared with traditional paper-based materials, multimedia information is more vivid and interesting and, thus, facilitates learning among older adults^24^. Studies have explored the effects of computer- and Internet-based education programs, of which 44.4% include integrated multimedia information, on patients with early prostate cancer and have compared the results to those of other treatment methods^14,25,26^. The results showed that Internet- and computer-based education programs can improve the knowledge, self-efficacy, and emotional support of patients with prostate cancer. Further high-quality research, however, is needed to verify the effectiveness of these programs^25^.

In the past few years, studies have investigated the efficacy of providing information through mobile apps^27^ or educational programs through the Internet^14,15,26,28^ to patients with early-stage prostate cancer in Western countries by using single-group^14^ and two-group methods^15,26–28^. The results indicate that such interventions improved patients’ QOL in terms of physical and social dimensions^14^, psychological distress^28^, fatigue, insomnia^27^, urinary symptoms^15^, and diversion coping^26^. The effect of such interventions on self-efficacy was not supported^15,26,28^, and the results for positive thinking were unclear. Overall, however, few studies have investigated multimedia-based information programs for patients with prostate cancer who are undergoing hormone therapy only^25,29^, and there is a lack of research on the efficacy of multimedia-based information programs in terms of positive thinking, QOL, social support, and self-efficacy among such patients. Therefore, further research is needed to understand the efficacy of information and education programs on prostate cancer patients undergoing hormone therapy, with a particular focus on self-efficacy and positive thinking.

### Theoretical foundation and frameworks

Systematic reviews have shown that interventions that use social cognitive theory as the framework can effectively increase the physical activity of cancer patients and help them to adopt a healthy diet^30^ and improve their QOL^31^. Social cognitive theory focuses on the interaction of individual cognition and environmental factors on learning. Based on this theory, the factors that affect individual behavior include individual cognitive, social environmental, and supportive behavioral factors.

The individual cognitive factors concern knowledge, self-efficacy, collective effective efficacy, and outcome expectations. Knowledge means that an individual can understand the advantages, disadvantages, and related information that are needed to engage in or modify behavior. Knowledge alone, however, is not sufficiently effective to enable individuals to engage in or modify behaviors^32,33^. Self-efficacy refers to an individual’s belief that he or she can achieve a goal. It is an important factor in deciding whether an individual should modify his or her behavior or adopt a new behavior. Therefore, improving an individual’s self-efficacy is critical to behavioral change. When individuals believe that their behavior can produce the expected outcomes, and they are capable of achieving a goal, they will take action and strive to reach the goal. Performance accomplishment, vicarious experience, verbal persuasion, and emotional arousal are four types of resource channels that can help individuals to improve their self-efficacy. Moreover, support and encouragement from others also can help individuals to form new behaviors or modify their current behaviors^32,33^. Therefore, when providing information to patients with prostate cancer, it is very important to increase their self-efficacy and social support.

The social-cognitive transition model of adjustment posits that individuals have an assumptive world, which includes assumptions and knowledge^34^. The assumptive world is formed by the individual’s interactions with the social environment during the development process. Assumptions are strengthened when the events experienced by the individual meet that person’s expectations. When the event does not meet expectations, however, stress will result, and the individual will need to modify his or her assumptions. To modify one’s assumptive world, the individual may engage in a positive transition, changing values or priorities, and this process may lead to healthy development or post-traumatic growth. If, instead, the individual engages in a negative transition, he or she loses motivation and self-confidence and becomes hopeless and depressed^34^. In the process of modifying the assumptive world through a positive transition, self-efficacy and social support are critical factors^34^, and research has revealed associations between social support, self-efficacy, positive thinking, and QOL of patients^17,21^.

In this study, the multimedia-based hormone therapy information program (HTIP) was developed based on social cognitive theory^32,33^ and the social cognitive transition model of adjustment^34^. The aim of the present study is to examine the feasibility and acceptability of HTIP as determined by the recruitment rate, retention rate, and satisfaction with the program among patients with prostate cancer. The preliminary efficacy of a multimedia-based HTIP in regard to (1) improving patients’ positive thinking and QOL (general QOL, prostate cancer-specific QOL, and hormone symptoms and distress, all of which are primary outcomes); and (2) increasing patients’ social support and self-efficacy, which are secondary outcomes.

## Methods

### Design

A prospective experimental study design was used. The data of patients who satisfied the inclusion criteria were collected through convenience sampling in the urology outpatient department of Linkou Chang Gung Memorial Hospital and Keelung Chang Gung Memorial Hospital, Taiwan, from May 2019 to January 2021. Patients who completed the pre-test questionnaire were randomly assigned to the multimedia information group (MIG) or the control group (CG), with an allocation ratio of 1:1. Patients in the MIG participated in a multimedia-based HTIP once a week for 6 weeks and received routine care. Patients in the CG received routine care. Data were collected from both groups at 8 and 12 weeks after the pre-test.

The design of this study was based on an earlier study^11^ and conditioned on the feasibility of the parameters of the present study, for which the intervention dose was set as once per week for 6 weeks. Chinese individuals over 70 years old are generally not familiar with the use of computers and Internet resources. Hence, media-based information courses were delivered through an instant messaging application on a smartphone, with which these persons were usually more comfortable. In accordance with the patient follow-up schedule, during which individuals received monthly hormone therapy injections on return to the clinic every 4 weeks, and considering relevant literature^11^, we also devised two post-tests with a 4-week interval between them. Post-test 1 was administered 8 weeks and post-test 2 was administered 12 weeks after the pre-test.

### Participants and sample size

The aim of the program is to enable patients to learn in an encouraging and supportive environment to enhance their cognitive, social environmental, and supportive behavioral factors. The patient’s partner or significant other (hereafter referred to as a cohabitant) also was invited to participate in the study and to learn with the patient. Cohabitants can provide support and care of patients in their daily life. Therefore, the study included patients who were (1) diagnosed with prostate cancer and underwent hormone therapy only; (2) living with their partner or significant other and had agreed to learn together; (3) able to communicate; and (4) able to connect to the Internet and had a smartphone. Those who (1) received radiotherapy, radical prostatectomy, or other treatments for prostate cancer; (2) suffered from mental illness; or (3) had an Eastern Cooperative Oncology Group Performance Status equal to or above 2^35^ were excluded from the study.

To our knowledge, no study with a two-group study design has been used to explore the efficacy of a multimedia-based information program for improving QOL, positive thinking, self-efficacy, or social support for patients with prostate cancer who are undergoing hormone therapy. Thus, the current study relied on the results of an eight-week multimedia information intervention for patients with early prostate cancer to estimate the number of participants needed^36^. In Loiselle et al.’s study, the scores of the 36-item Short Form Health Survey (SF-36) for the mental QOL of patients with prostate cancer were as follows: experimental group: 58.7 points (SD = 8.7) and control group: 53.0 points (SD = 11.1)^36^. Package *longpower* in R v.4.2.2 was used on these data to estimate the number of samples^37^. The statistical tests used included a generalized estimating equation (GEE), with the statistical power set at 0.80, *α* set at 0.05, and three time points for measurements. The result shows that the total number of participants required was 70, with at least 35 patients for each group. Considering a 10% discontinuation rate^38^, a minimum of 40 participants was thus indicated for each group.

### Procedures and data collection

Researchers were trained in the procedures and data collection before conducting the research. During the study period, all methods were performed in accordance with the relevant guidelines and regulations. Patients were recruited from the hospitals’ urology outpatient department. Those who met the inclusion criteria were informed of the purpose of the study and invited to participate. After patients signed the informed consent form, pre-test data were collected. Because the participants were not familiar with how to complete an online questionnaire, paper questionnaires were used for each of the three instances of data collection. The pre-test questionnaire was completed in a private place when patients visited the outpatient department for follow-up.

The principal investigator obtained an assignment code through a random-number generator and placed each code into an opaque envelope. After the pre-test, the researcher opened the envelopes and used the randomly obtained code to assign the patients to the MIG or the CG. For the MIG, the researcher confirmed that the instant messaging application was installed on the participants’ smartphones. An information program guidebook was provided to patients in the MIG and their cohabitants. The patients and their cohabitants confirmed that they could successfully link to the multimedia information courses, using the instant messaging application. They also confirmed that they could use the instant messaging application to make calls and send messages.

The multimedia information courses were distributed to the patients and cohabitants via an instant messaging application; a text message was sent to their mobile phone to remind them to view the content each week. The intervenor confirmed that the patients and cohabitants watched the multimedia information courses and set an appointment with them for after-class consultation and reinforcement, once a week for 6 weeks, during which the intervenor called the patient for after-class consultation and reinforcement.

Post-test 1 (Time 1) and Post-test 2 (Time 2) paper questionnaires were used and were completed at 8 and 12 weeks, respectively, after the pre-test for the two groups. The post-test questionnaire was completed when patients returned to the outpatient department for follow-up. With their consent, a few patients were sent paper questionnaires because the in-hospital questionnaire administration time did not match the schedule of the patients’ hospital visits.

In this study, the multimedia-based information program and instant messaging application were implemented without any interaction or communication between the intervenor and the CG. Furthermore, aside from visiting the hospital outpatient clinic for necessary follow-up due to illness, participants spent the remainder of their time either at home or in the community. There were no specific interactions between the MIG and the CG during the brief outpatient waiting periods. Additionally, the outpatient medical staff remained unaware of which patients were part of this study and their respective groups. In consideration of research ethics, this study did not impose restrictions on the MIG and the CG regarding additional self-learning of self-care skills. However, the MIG was verbally informed that the learning materials and content should not be shared with other patients or individuals outside their families. Following the conclusion of the experiment, this study provided the developed multimedia information courses and information program guidebook to the CG for their own use and learning.

### Intervention

**Figure 1:**
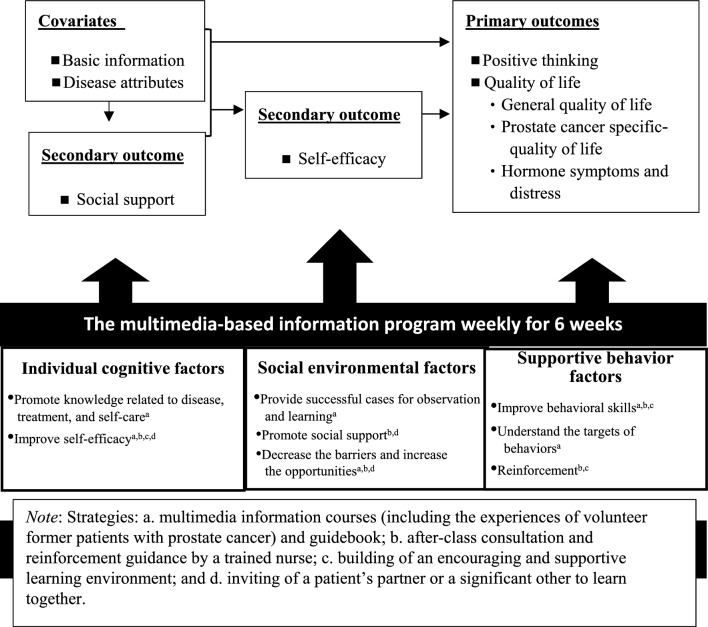
Conceptual framework of the multimedia-based information program.

A multimedia-based HTIP, to be implemented once a week for 6 weeks, was developed. The program is based on the relevant literature^2,5,11–13,17,21,39–41^, social cognitive theory^32,33^, and a social cognitive transition model of adjustment^34^. The multimedia-based HTIP consists of three parts: multimedia information courses, an information program guidebook, and after-class consultation and reinforcement (Fig. 1).

#### Multimedia information courses

Previous qualitative studies have shown that patients with prostate cancer who are receiving hormone therapy experience low energy, poor sleep quality, psychological distress, negative emotions, and inability to participate in leisure activities due to distress^5^. In accordance with these findings, six topics were covered in the multimedia information courses: introduction to hormone therapy and the multimedia program, mindfulness-based stress reduction, enhancement of health, relief of stress, positive thinking, and preventing and managing fatigue (Table 1). The material was written by the principal investigator. The content was then examined by experts from a variety of related fields, including urological oncology, psychology, psychiatry, mindfulness-based stress reduction, nursing, and exercise. After revising the content in accordance with the experts’ feedback, we produced six multimedia information courses that used animation, pictures, speech, and music. In addition, former patients with prostate cancer were invited to share their experiences and record relevant videos for integration into the multimedia information courses. Then, based on the patients’ feedback in regard to face validity, the multimedia information courses were adjusted in terms of such items as the color and size of subtitles.

**Table 1:** Topics and outline of the multimedia information course.

Week	Topic	Outline
1	Introduction to hormone therapy and the multimedia information program	Anatomical structure and functions of the prostate; hormone therapy; possible side effects of hormone therapy and methods for their prevention and mitigation; application of the multimedia hormone therapy information program; and patients’ sharing of experiences
2	Mindfulness-based stress reduction	What is mindfulness-based stress reduction and its benefits? Preparation for the mindfulness-based stress reduction exercise; how to perform mindfulness-based stress reduction; practicing mindfulness-based stress reduction exercises; recording practice status
3	Enhancement of health	What are the benefits of exercise? Opportune moments for exercise; assessment; points for attention and skills (aerobic, resistance, and flexibility exercises); demonstration
4	Relief of stress	What is stress? Causes of stress, body’s response to stress; stress management strategies, e.g., planting vegetables/flowers, training in self-confidence skills, engaging in appropriate social activities, having social resources; and patients’ sharing of experiences
5	Positive thinking	What is positive thinking? Looking at things from different perspectives; how to avoid automatic negative thinking and creating negative emotions; practice to avoid looking at things from an unreasonable perspective; practice to look at things from a reasonable perspective; record practice status; and patients’ sharing of experiences
6	Preventing and managing fatigue	What is cancer-related fatigue and its possible causes? Methods for evaluating cancer-related fatigue; skills for preventing or relieving cancer-related fatigue; monitor and record fatigue level; arrange and adjust daily life to save energy, exercise, stress adjustment, and develop good sleeping habits; and patients’ sharing of experiences

#### Information program guidebook

The program guidebook included the following: (1) theoretical basis and implementation of the program; (2) instructions on how to access the multimedia information courses, using an instant messaging application; (3) instructions on how to make calls and send messages via an instant messaging application; (4) the key content of each unit of the multimedia information courses; and (5) the recording sheets of after-class tasks.

#### After-class consultation and reinforcement

A trained nurse with 10 years of urological experience served as the intervenor. The primary tasks of the intervenor included: (1) addressing the participants’ questions and concerns regarding the multimedia information content; (2) encouraging the participants to apply the skills they learned in the courses to their daily life (an after-class task); (3) understanding the participants’ experiences and difficulties in applying the skills they learned and providing timely clarification; (4) discussing the previous week’s after-class task; and (5) recording the participants’ questions about Tasks 1, 3, and 4. The patients and cohabitants were guided by the intervenor to complete this activity using the following questions: Do you have any questions about the multimedia information this week? How did the after-class tasks go last week? How was the patient’s health condition last week? Did the patient experience any physical or mental discomfort? How did you deal with it? Were there any problems with self-care?

### Routine care

All prostate cancer patients undergoing hormone therapy received verbal health education, covering various aspects of their illness, treatment, and self-care precautions. This included guidance on topics such as diet, exercise, smoking cessation, and common treatment side effects (e.g., hot flashes, osteoporosis, muscle weakness, and potential increase in body fat percentage). Additionally, individualized information was offered to address each patient's specific health concerns.

### Measures

Primary and secondary outcome variables were measured at pre-test and at 8 and 12 weeks after. Basic information and disease attributes were collected at pre-test, and satisfaction with the program was assessed at 8 weeks after. The recruitment rate and retention rate were also calculated.

#### Primary outcome variables

The *Positive Thinking Scale-Chinese version* consists of 18 questions, with a total score that ranges from 18 to 90, with higher scores as indicating more positive thinking. Both the original scale and the Chinese version have acceptable reliability and validity^17–20^.

The *Functional Assessment of Cancer Therapy (FACT)-Prostate (P)-Chinese version* assesses well-being and includes a prostate cancer subscale. It provides scores that indicate general QOL (FACT-G) and QOL as related to prostate cancer (FACT-P)^42–44^. The scale comprises 39 questions, with scores that range from 0 to 156; higher scores indicate better QOL. The scale has acceptable reliability and validity^44^. Clinically meaningful results can be achieved when the FACT-G score changes by 6–7^45^, and the FACT-P score changes by 6 to 10 points^46^.

The *Expanded Prostate Cancer Index Composite-Chinese version* comprises four subscales (i.e., urinary, bowel, sexual, and hormone)^47^. The scores of the four subscales range from 0 to 100, and higher scores indicate better status of a dimension. The original scale exhibits good reliability and validity^48^. In the Chinese scale, the Cronbach’s *α* of internal consistency ranges from 0.70 to 0.92^47^. In this study, the hormone subscale was used to measure the hormone symptoms and distress of patients with prostate cancer.

#### Secondary outcome variables

The *Social Support Scale-Chinese version* was used to measure the support provided to patients by non-professionals. It comprises 16 questions, with scores that range from 0 to 48. Higher scores denote more support provided to patients by their family, relatives, and friends. The scale has acceptable content validity, and the Cronbach’s *α* of internal consistency was 0.95^49,50^.

The *General Self-Efficacy Scale-Chinese version* comprises 10 questions, with scores that range from 10 to 40, for which higher scores indicate higher self-efficacy. The scale has acceptable validity and reliability^51–53^.

#### Basic information and disease attributes

A *data sheet* was used to collect basic information and disease attributes of the patients. Basic information included age, educational level, marital status, religious beliefs, occupational status, annual household income, residential status, body mass index (BMI), and perceived health status. Disease attributes included the time since diagnosis, most recent concentration of serum prostate-specific antigen (PSA), cancer staging, history of past illness, and types of hormone therapy. Disease attributes were obtained from medical charts and confirmed by the participants.

#### Feasibility and acceptability

In this study, feasibility was assessed by recruitment rate and retention rate, and acceptability was assessed by satisfaction with the program. The recruitment rate was calculated as [(total number of participants recruited during study period/total number of hospitals) divided by months of duration of recruitment]^54^. Satisfaction with the multimedia-based HTIP was assessed using a questionnaire that comprised five questions, developed based on a previous study^11^. The score of each item ranged from 0 to 100, and higher scores indicated higher satisfaction or agreement.

### Ethical considerations

This study was reviewed and approved by the Institutional Review Board of the hospital. The researchers complied with all research ethical norms. All patients provided informed consent and had the right to withdraw from the study at any point without affecting their treatment.

### Ethical approval and consent to participate

The study project was reviewed and approved by Chang Gung Medical Foundation Institutional Review Board (No. 201602024B0C501). All participants verbally agreed to participant this study and provided written informed consent.

### Data analysis

Statistical analysis was performed using IBM SPSS Statistics 22.0. software (Armonk, NY, USA). A two-tailed *p*-value of less than 0.05 was considered statistically significant. The study had no missing data and estimated the outcomes of the program for all participants (*n* = 76).

The homogeneity of basic attributes, disease variables, positive thinking, QOL, social support, and self-efficacy between the MIG and CG in the pre-test was confirmed through independent sample *t*-tests, chi-square tests, and Fisher’s exact test. Basic attributes and disease variables that exhibited statistically significant differences between groups were included in the model for the evaluation of the intervention efficacy (covariate). The covariates were controlled for using a generalized estimating equation (GEE) to understand the preliminary efficacy of the intervention program in improving positive thinking, QOL, social support, and self-efficacy of patients with prostate cancer. Furthermore, this study employed GEE to assess each variable within the MIG and the CG separately, aiming to determine whether the scores in post-tests 1 and 2 exhibited significant changes compared to the pre-test scores. Considering patients’ disease progression following their initial hormone therapy^3^ and the disparities in basic attributes between MIG and CG during the pre-test, this study focused on estimating the program outcomes within a subgroup consisting of patients diagnosed with prostate cancer within two years (n = 48). Furthermore, a more detailed subgroup analysis was conducted to assess the intervention efficacy in prostate cancer patients diagnosed within two years, who were not employed and had an annual household income less than NT$500,000 (*n* = 32). Finally, the Cohens’ *d* (formula = [mean in MIG − mean in CG]/SDpooled) was calculated to understand the effect size of the difference between MIG and CG at pre-test, post-test 1, and post-test 2^55^.

## Results

### Recruitment

**Figure 2:**
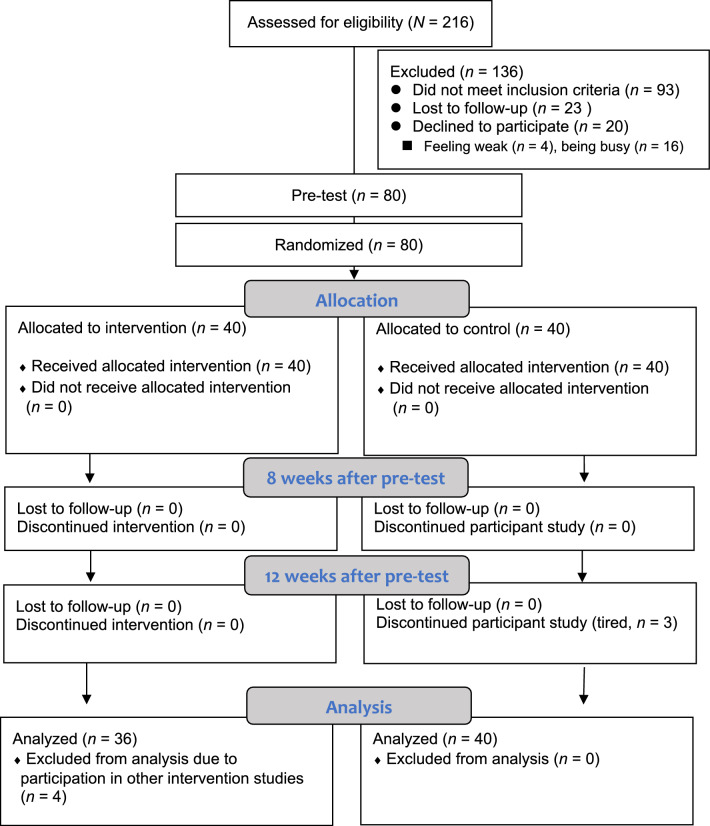
Flowchart of participant recruitment and follow-up.

A total of 216 patients were screened. Of these, 100 patients satisfied the inclusion criteria, and 80 patients provided informed consent to participate in this study (average recruitment rate = 2 participants from each hospital per month; 80% enrollment rate). Twenty patients declined to participate in the research because they felt too weak (*n* = 4) or were unavailable (*n* = 16). After completing the pre-test questionnaire, 80 patients were randomly assigned to the MIG (*n* = 40) or the CG (*n* = 40). Three patients in the CG, after completing the questionnaire at 8 weeks after pre-test, withdrew from the study due to feeling weak (96.3% retention rate, 77 completers out of 80 participants). Four patients in the MIG declared that they had participated in another intervention study and, thus, were excluded from the data analysis to avoid data interference. Ultimately, 76 patients were included in the statistical analysis (Fig. 2).

### Homogeneity of pre-test data

In terms of the total sample (*N* = 76), the results of the homogeneity tests of the MIG and the CG in terms of the pre-test’s basic information and disease attributes and outcome variables showed that the two groups were not homogeneous in terms of occupational status (*p* < 0.05), annual household income (*p* < 0.05), or months since diagnosis (*p* < 0.01). Other variables were not significantly different between the two groups, which indicated that the scores of the two groups, including age, educational level, marital status, religious belief, residential status, BMI, perceived health status, recent concentration of serum PSA, cancer stage, history of past illness, types of hormone therapy, positive thinking, general QOL, prostate cancer-specific QOL, hormone symptoms and distress, social support, and self-efficacy, were similar (all *p* > 0.05; Table 2).

**Table 2:** Demographic information and clinical variables at time of pre-test evaluation.

Variable	Entire sample (*N* = 76)			Diagnosed within 2 years (*n* = 48)		
	MIG (*n* = 36)	CG (*n* = 40)	χ^2^/*t*	MIG (*n* = 28)	CG (*n* = 20)	χ^2^/*t*
	*n* (%) [Mean ± SD]	*n* (%) [Mean ± SD]		*n* (%) [Mean ± SD]	*n* (%) [Mean ± SD]	
Age (years)	[73.22 ± 7.54]	[76.83 ± 8.33]	1.97	[73.75 ± 7.52]	[77.35 ± 9.66]	1.45
Educational level			0.89			0.20
< Elementary school	15 (41.7)	21 (52.5)		13 (45.4)	8 (40.0)	
≥ Junior high school	21 (58.3)	19 (47.5)		15 (53.6)	12 (60.0)	
Marital status			–^a^			–^a^
Married/cohabitating	30 (83.3)	36 (90.0)		22 (78.6)	19 (95.0)	
Divorced/widowed	6 (16.7)	4 (10.0)		6 (21.4)	1 (5.0)	
Religious beliefs			1.26			–^a^
No	8 (22.2)	5 (12.5)		8 (28.6)	3 (15.0)	
Yes	28 (77.8)	35 (87.5)		20 (71.4)	17 (85.0)	
Occupational status			6.12*			–^a^**
No	27 (75.0)	38 (95.0)		20 (71.4)	20 (100.0)	
Yes	9 (25.0)	2 (5.0)		8 (28.6)	0 (0.0)	
Annual household income			4.11*			–^a^
< NT$500,000	30 (83.3)	25 (62.5)		23 (82.1)	16 (80.0)	
≥ NT$500,000	6 (16.7)	15 (37.5)		5 (17.9)	4 (20.0)	
Residential status			3.28			4.61
Other residential status	9 (25.0)	6 (15.0)		9 (32.1)	2 (10.0)	
Live with partner only	5 (13.9)	12 (30.0)		5 (17.9)	8 (40.0)	
Live with partner and children with/without grandchildren	22 (61.1)	22 (55.0)		14 (50.0)	10 (50.0)	
Body mass index			0.65			0.29
18.5–24 (Normal)	13 (36.1)	11 (27.5)		9 (32.1)	5 (25.0)	
< 18.5 (Abnormal)	23 (63.9)	29 (72.5)		19 (67.9)	15 (75.0)	
Perceived health status	[70.17 ± 11.66]	[71.88 ± 14.84]	0.55	[68.79 ± 12.27]	[68.50 ± 14.70]	− 0.07
Time since diagnosis (months)	[16.34 ± 22.15]	[47.58 ± 51.44]	3.50**	[6.66 ± 6.50]	[9.35 ± 8.10]	1.27
Recent concentration of serum PSA	[84.18 ± 245.39]	[23.49 ± 73.78]	1.43	[108.08 ± 274.58]	[24.17 ± 44.81]	− 1.59
Cancer stage			0.28			0.01
Stage I–III	8 (22.2)	11 (27.5)		8 (28.6)	6 (30.0)	
Stage IV	28 (77.8)	29 (72.5)		20 (71.4)	14 (70.0)	
History of past illness			–^a^			0.04
No	5 (13.9)	4 (10.0)		5 (17.9)	4 (20.0)	
Yes	31 (86.1)	36 (90.0)		23 (82.1)	16 (80.0)	
Types of hormone therapy			6.08			1.51
Orchiectomy	0 (0.0)	2 (5.0)		0 (0.0)	1 (5.0)	
Medical castration (injection only)	16 (44.4)	17 (42.5)		15 (53.6)	11 (55.0)	
Medical castration (injection and oral)	20 (55.6)	17 (42.5)		13 (46.4)	8 (40.0)	
Orchiectomy + medical castration	0 (0.0)	4 (10.0)		0 (0.0)	0 (0.0)	
Positive thinking	[67.17 ± 7.07]	[67.23 ± 7.42]	0.03	[66.36 ± 7.59]	[66.35 ± 7.53]	< − 0.01
General QOL	[79.86 ± 13.95]	[79.18 ± 15.42]	− 0.20	[76.6.79 ± 12.82]	[78.50 ± 11.75]	0.47
Prostate cancer-specific QOL	[116.39 ± 18.44]	[115.00 ± 20.92]	− 0.31	[112.21 ± 17.36]	[113.55 ± 16.87]	0.27
Hormone symptoms and distress	[85.67 ± 14.48]	[84.66 ± 15.45]	− 0.29	[84.17 ± 14.83]	[86.82 ± 12.87]	0.64
Social support	[34.69 ± 8.20]	[35.43 ± 9.56]	0.36	[34.50 ± 7.97]	[36.10 ± 8.04]	0.68
Self-efficacy	[29.44 ± 5.84]	[29.90 ± 5.32]	0.36	[28.21 ± 5.19]	[30.70 ± 5.08]	1.65

^a^Fisher’s exact test.

*p < 0.05, **p < 0.01.

*CG* control group, *MIG* multimedia information group, *QOL* quality of life, *PSA* prostate-specific antigen, *SD* standard deviation.

In terms of the sample who were diagnosed within two years (*n* = 48), the results for the homogeneity tests of the MIG and the CG in terms of the pre-test’s basic information and disease attributes as well as the outcome variables showed that the two groups were not similar in terms of occupational status (*p* < 0.05). Other variables were not significantly different between the two groups, which indicated that the scores of the two groups were similar (all *p* > 0.05; Table 2).

### Primary outcomes

For the overall sample, variations in outcome scores between the MIG and the CG across different time points were observed. The positive thinking scores for MIG (*B* = 1.67, *B* = 1.33; all *p* > 0.05), along with general QOL scores (*B* = 1.03, *B* = 0.58; all *p* > 0.05) and prostate cancer-specific QOL scores (*B* = 1.86, *B* = 1.81; all *p* > 0.05) in weeks 8 and 12, demonstrated improvements compared to the pre-test scores. Additionally, the MIG's hormone symptoms and distress scores were higher than the pre-test score in the 8th week but lower than the pre-test scores in the 12th week (*B* = 1.58, *B* = − 2.27; all *p* > 0.05). Nevertheless, these changes did not achieve statistical significance. In contrast, the CG exhibited a decline in positive thinking scores (*B* = − 0.10, *B* = − 0.24; all *p* > 0.05) in weeks 8 and 12 compared to the pre-test. Furthermore, in both 8th and 12th weeks, CG's general QOL scores (*B* = 0.10, *B* = 0.63; all *p* > 0.05), prostate cancer-specific QOL scores (*B* = 1.75, *B* = 1.37; all *p* > 0.05), as well as hormone symptoms and distress scores (*B* = 1.53, *B* = 1.75; all* p* > 0.05) were all higher than the pre-test scores. However, similar to the MIG, none of these changes reached statistical significance. In terms of score differences between the two groups, after we controlled for occupational status, annual household income, and time since diagnosis, at 8 and 12 weeks, the MIG scored higher than the CG in positive thinking (*B* = 1.77, *B* = 1.61; all *p* > 0.05) and prostate cancer-specific QOL (*B* = 0.11, *B* = 0.42; all *p* > 0.05), but the differences were not statistically significant. The MIG scored higher than the CG for general QOL and hormone symptoms and distress at 8 weeks (*B* = 0.93, *B* = 0.04; all *p* > 0.05) and lower than did the CG at 12 weeks (*B* = − 0.07, *B* = − 3.99; all *p* > 0.05), but, again, the differences were not statistically significant (Table 3).

**Table 3:** Evaluation of the preliminary outcomes of the program for patients who received hormone therapy.

Outcome	MIG (*n* = 36)^a^		CG (*n* = 40)^a^		Cohen’s *d*^b^	Generalized estimating equation^c^		
						Time	Group × time interaction effects	
	Mean	SD	Mean	SD		*B* (*SE*)	*B* (*SE*)	95% CI
Primary outcomes								
Positive thinking								
Pre-test	67.17	7.07	67.23	7.42	< − 0.01			
8 weeks	68.83	7.43	67.13	8.06	0.22	− 0.10 (0.82)	1.77 (1.27)	− 0.73, 4.26
12 weeks	68.50	8.73	66.62	7.57	0.23	− 0.28 (0.77)	1.61 (1.25)	− 0.83, 4.06
General QOL								
Pre-test	79.86	13.95	79.18	15.42	0.05			
8 weeks	80.89	13.84	79.28	19.54	0.10	0.10 (2.01)	0.93 (2.77)	− 4.50, 6.35
12 weeks	80.44	14.63	80.46	16.30	< − 0.01	0.66 (1.73)	− 0.07 (2.48)	− 4.93, 4.79
Prostate cancer-specific QOL								
Pre-test	116.39	18.44	115.00	20.92	0.07			
8 weeks	118.25	17.69	116.75	25.24	0.07	1.75 (2.58)	0.11 (3.53)	-6.81, 7.04
12 weeks	118.19	18.57	117.35	21.92	0.04	1.39 (2.35)	0.42 (3.21)	-5.88, 6.71
Hormone symptoms and distress								
Pre-test	85.67	14.48	84.66	15.45	0.07			
8 weeks	87.25	12.91	86.19	15.29	0.08	1.53 (2.34)	0.04 (2.88)	− 5.61, 5.69
12 weeks	83.40	14.71	86.79	14.55	− 0.23	1.72 (2.21)	− 3.99 (3.18)	− 10.23, 2.24
Secondary outcomes								
Social support								
Pre-test	34.69	8.20	35.43	9.56	− 0.08			
8 weeks	34.89	9.04	34.33	9.38	0.06	− 1.10 (1.00)	1.29 (1.66)	− 1.95, 4.54
12 weeks	34.44	10.00	33.49	9.15	0.01	− 1.36 (1.50)	1.11 (2.05)	− 2.90, 5.12
Self-efficacy								
Pre-test	29.44	5.84	29.90	5.32	− 0.08			
8 weeks	29.08	4.00	30.18	5.16	− 0.24	0.28 (0.65)	− 0.64 (0.97)	− 2.53, 1.26
12 weeks	30.47	5.69	29.70	5.02	0.14	− 0.07 (0.77)	1.10 (1.20)	− 1.25, 3.44

Cases included in data analysis at pre-test, 8 weeks, and 12 weeks: MIG—36, 36, and 36, respectively, CG—40, 40, and 37, respectively.

^a^Generalized estimating equations were employed to assess changes over time, with the pre-test serving as the reference group. All *p* values > 0.05.

^b^Cohen’s *d* = (Mean in MIG − Mean in CG)/SDpooled.

^c^Control covariates: annual household income, occupational status, and time since diagnosis.

*CG* control group, *CI* confidence interval, *MIG* multimedia information group, *QOL* quality of life, *SD* standard deviation, *SE* standard error.

Further analysis was conducted on the subgroup of patients who were diagnosed with prostate cancer within two years (MIG = 28, CG = 20). In terms of the outcome scores for both the MIG and the CG groups at different time points, it’s noteworthy that in weeks 8 and 12, MIG exhibited higher scores compared to their respective pre-test levels in positive thinking (*B* = 1.69, *B* = 1.69; all* p* > 0.05), general QOL (*B* = 2.59, *B* = 2.14; all *p* > 0.05), and prostate cancer-specific QOL (*B* = 4.14, *B* = 4.14; all *p* > 0.05). Additionally, the MIG’s hormone symptoms and distress scores were higher than the pre-test score in the 8th week but lower than that in the 12th week (*B* = 2.04, *B* = − 2.35; all *p* > 0.05). The positive thinking scores for CG during both weeks 8 and 12 exceeded that of the pre-test (*B* = 1.00, *B* = 1.46; all *p* > 0.05). However, it is worth noting that the CG's general QOL (*B* = − 2.90, *B* = − 1.50; all *p* > 0.05), prostate cancer-specific QOL (*B* = − 1.60, *B* = − 1.07; all *p* > 0.05), as well as hormone symptoms and distress (*B* = − 1.59, *B* = − 1.65; all *p* > 0.05) scores during weeks 8 and 12 were all lower than their respective pre-test scores, although none of these changes achieved statistical significance. In terms of score differences between the two groups, after we controlled for occupational status, at 8 and 12 weeks, the MIG scored lower than the CG in positive thinking (*B* = − 0.11, *B* = − 0.54; all *p* > 0.05), but the differences were not statistically significant. In terms of general QOL and prostate cancer-specific QOL, the MIG scored higher than the CG at 8 (*B* = 5.79, *B* = 6.10; all *p* > 0.05) and 12 weeks (*B* = 3.73, *B* = 5.37; all *p* > 0.05), but the differences were not statistically significant. The MIG scored higher than the CG in terms of hormone symptoms and distress at 8 weeks (*B* = 3.70; *p* > 0.05) but lower at 12 weeks (*B* = − 0.92; *p* > 0.05); however, again, the differences were not statistically significant (Table 4).

**Table 4:** Evaluation of the preliminary outcomes of the program for patients who were diagnosed with prostate cancer within two years.

Outcome	MIG (*n* = 28)^a^		CG (*n* = 20)^a^		Cohen’s *d*^b^	Generalized estimating equation^c^		
						Time	Group × time interaction effects	
	Mean	SD	Mean	SD		*B* (*SE*)	*B* (*SE*)	95% CI
Primary outcomes								
Positive thinking								
Pre-test	66.36	7.59	66.35	7.53	< 0.01			
8 weeks	67.25	6.40	67.35	7.94	− 0.01	1.00 (0.96)	− 0.11 (1.30)	− 2.65, 2.44
12 weeks	67.25	8.16	66.89	8.01	0.04	1.43 (0.79)	− 0.54 (1.19)	− 2.87, 1.79
General QOL								
Pre-test	76.79	12.82	78.50	11.75	− 0.14			
8 weeks	79.68	13.46	75.60	17.06	0.07	− 2.90 (2.92)	5.79 (3.72)	− 1.49, 13.08
12 weeks	78.96	14.12	76.56	14.48	0.17	− 1.55 (2.47)	3.73 (3.25)	− 2.63, 10.09
Prostate cancer-specific QOL								
Pre-test	112.21	17.36	113.55	16.87	− 0.08			
8 weeks	116.71	17.81	111.95	21.41	0.24	− 1.60 (3.53)	6.10 (4.55)	− 2.81, 15.01
12 weeks	116.46	18.22	111.89	20.40	0.24	− 1.12 (3.28)	5.37 (4.14)	− 2.74, 13.48
Hormone symptoms and distress								
Pre-test	84.17	14.83	86.82	12.87	− 0.19			
8 weeks	86.28	12.97	85.23	15.46	0.07	− 1.59 (2.68)	3.70 (3.33)	− 2.82, 10.22
12 weeks	81.74	14.83	86.36	14.83	− 0.31	− 1.52 (2.72)	− 0.92 (3.95)	− 8.65, 6.82
Secondary outcomes								
Social support								
Pre-test	34.50	7.97	36.10	8.04	− 0.20			
8 weeks	34.39	8.39	35.85	7.80	− 0.18	− 0.25 (1.02)	0.14 (1.80)	− 3.39, 3.68
12 weeks	33.79	9.41	33.94	6.38	− 0.02	− 1.24 (1.88)	0.53 (2.45)	− 4.28, 5.33
Self-efficacy								
Pre-test	28.21	5.19	30.70	5.08	− 0.48			
8 weeks	28.39	3.76	29.70	4.68	− 0.31	− 1.00 (0.76)	1.18 (0.98)	− 0.73, 3.09
12 weeks	29.64	5.50	29.00	4.09	0.13	− 1.28 (0.78)	2.71 (1.27)*	0.22, 5.20

Cases included in data analysis at pre-test, 8 weeks, and 12 weeks: MIG—28, 28, and 28, respectively, CG—20, 20, and 18, respectively.

^a^Generalized estimating equations were employed to assess changes over time, with the pre-test serving as the reference group. All *p* values > 0.05.

^b^Cohen’s d = (Mean in MIG − Mean in CG)/SDpooled.

^c^Control covariate: occupational status. *p < 0.05.

*CG* control group, *CI* confidence interval, *MIG* multimedia information group, *QOL* quality of life, *SD* standard deviation, *SE* standard error.

Further analysis was conducted on the subgroup of patients who were diagnosed with prostate cancer within two years, were not employed and had an annual household income less than NT$500,000. The outcome scores for both the MIG and CG groups exhibited changes at different time points. In both weeks 8 and 12, MIG demonstrated higher scores in positive thinking (*B* = 1.71, *B* = 1.06; all *p* > 0.05), general QOL (*B* = 2.59, *B* = 1.29; all *p* > 0.05), and prostate cancer-specific QOL (*B* = 4.65, *B* = 3.88; all *p* > 0.05) compared to their pre-test levels. The hormone symptoms and distress score for the MIG (*B* = 2.00, *B* = − 0.94; all *p* > 0.05) was higher than the pre-test level in the 8th week but decreased below the pre-test level in the 12th week. For the CG, positive thinking scores in both weeks 8 and 12 were higher than the pre-test score (*B* = 0.07, *B* = 1.09; all* p* > 0.05). However, CG's general QOL (*B* = − 2.53, *B* = − 1.10; all *p* > 0.05), prostate cancer-specific QOL (*B* = − 0.73, *B* = − 0.13; all *p* > 0.05), and hormone symptoms and distress (*B* = − 0.46, *B* = − 1.43; all *p* > 0.05) scores during weeks 8 and 12 were all lower than their respective pre-test scores, but none of these changes reached statistical significance. In terms of the disparities in scores between the two groups, in positive thinking, general QOL, prostate cancer-specific QOL, and hormone symptoms and distress, the MIG scored higher than the CG at 8 (*B* = 1.64, *B* = 5.12, *B* = 5.38, *B* = 2.46; all *p* > 0.05) and 12 weeks (*B* = 0.05, *B* = 2.53, *B* = 4.18, *B* = 0.39; all *p* > 0.05); however, the differences were not statistically significant (Table 5).

**Table 5:** Evaluation of the preliminary outcomes of the program for patients who were diagnosed with prostate cancer within two years, were not employment, and had low household income.

Outcome	MIG (*n* = 17)^a^		CG (*n* = 15)^a^		Cohen’s *d*^b^	Generalized estimating equation		
						Time	Group × time interaction effects	
	Mean	SD	Mean	SD		*B* (*SE*)	*B* (*SE*)	95% CI
Primary outcomes								
Positive thinking								
Pre-test	64.59	6.23	66.00	8.01	− 0.20			
8 weeks	66.29	5.93	66.07	8.55	0.03	0.07 (0.99)	1.64 (1.49)	− 1.28, 4.56
12 weeks	65.65	7.69	65.62	8.60	< 0.01	1.01 (1.04)	0.05 (1.51)	− 2.91, 3.01
General QOL								
Pre-test	76.88	13.62	76.27	10.82	0.05			
8 weeks	79.47	14.34	73.73	17.92	0.35	− 2.53 (3.49)	5.12 (4.90)	− 4.49, 14.73
12 weeks	78.18	14.73	74.31	14.63	0.26	− 1.24 (3.07)	2.53 (4.09)	− 5.48, 10.55
Prostate cancer-specific QOL								
Pre-test	112.76	18.94	110.07	15.90	0.15			
8 weeks	117.41	18.10	109.33	22.70	0.39	− 0.73 (4.12)	5.38 (6.00)	− 6.39, 17.15
12 weeks	116.65	18.99	108.77	20.92	0.39	− 0.30 (4.22)	4.18 (5.31)	− 6.23, 14.59
Hormone symptoms and distress								
Pre-test	84.63	16.98	85.00	13.95	− 0.02			
8 weeks	86.63	14.53	84.55	15.23	0.14	− 0.45 (2.99)	2.46 (4.29)	− 5.95, 10.87
12 weeks	83.69	14.62	85.14	16.01	− 0.09	− 1.33 (3.47)	0.39 (4.91)	− 9.22, 10.01
Secondary outcomes								
Social support								
Pre-test	34.00	8.60	35.07	6.80	− 0.14			
8 weeks	34.76	8.88	34.33	7.47	0.05	− 0.73 (1.04)	1.50 (2.36)	− 3.12, 6.12
12 weeks	34.71	9.10	33.54	7.16	0.14	− 0.08 (1.73)	0.78 (2.75)	− 4.61, 6.18
Self-efficacy								
Pre-test	27.65	5.04	29.80	4.55	− 0.45			
8 weeks	28.35	3.77	29.27	4.51	− 0.22	− 0.53 (0.80)	1.24 (1.13)	− 0.97, 3.45
12 weeks	29.65	5.89	28.23	4.00	0.28	− 0.97 (0.96)	2.97 (1.80)	− 0.55, 6.50

Cases included data analysis at pre-test, 8 weeks, and 12 weeks: MIG—17, 17, and 17, respectively, CG—15, 15, and 13, respectively.

^a^Generalized estimating equations were employed to assess changes over time, with the pre-test serving as the reference group. All *p* values > 0.05.

^b^Cohen’s *d* = (Mean in MIG − Mean in CG)/SDpooled. *p < 0.05, **p < 0.01.

*CG* control group, *CI* confidence interval, *MIG* multimedia information group, *QOL* quality of life, *SD* standard deviation, *SE* standard error.

### Secondary outcomes

For the entire sample, in terms of changes in outcome scores for both the MIG and CG at different time points, the MIG's social support score was higher than the pre-test score in the 8th week but declined below the pre-test level in the 12th week (*B* = 0.19, *B* = − 0.25; all *p* > 0.05). On the other hand, the MIG's self-efficacy score was lower than the pre-test score in week 8 but showed an increase above the pre-test level in week 12 (*B* = − 0.36, *B* = 1.03; all *p* > 0.05). For the CG, both the social support scores (*B* = − 1.10, *B* = − 1.36; all *p* > 0.05) in weeks 8 and 12 were lower than the pre-test score. Additionally, the CG’s self-efficacy scores (*B* = 0.28, *B* = 0.01; all *p* > 0.05) were higher than the pre-test score in both weeks 8 and 12, although none of these changes reached statistical significance. In terms of the score differences between the two groups after controlling for covariates, at 8 and 12 weeks, the MIG scored higher than the CG in social support (*B* = 1.29, *B* = 1.11; all *p* > 0.05), but the differences were not statistically significant. The scores for self-efficacy of the MIG were lower than those of the CG at 8 weeks (*B* = − 0.64; *p* > 0.05) and higher than those of the CG at 12 weeks (*B* = 1.10; *p* > 0.05); however, again, the differences were not statistically significant (Table 3).

Further analysis was conducted on the subgroup of patients who were diagnosed with prostate cancer within 2 years. Regarding changes in the outcome scores for both MIG and CG at different time points, the MIG’s social support scores in both weeks 8 and 12 were lower than the pre-test score (*B* = − 0.10, *B* = − 0.69; all *p* > 0.05). Conversely, the MIG’s self-efficacy scores in both weeks 8 and 12 were higher than the pre-test score (*B* = 0.35, *B* = 1.72; all *p* > 0.05). In contrast, the CG's scores for both social support (*B* = − 0.25, *B* = − 1.36; all *p* > 0.05) and self-efficacy (*B* = − 1.00, *B* = − 1.23; all *p* > 0.05) in weeks 8 and 12 were lower than the pre-test level, but none of these changes reached statistical significance. In terms of the score differences between the two groups, after we controlled for covariate, the MIG scored higher than the CG in social support at 8 (*B* = 0.14; *p* > 0.05) and 12 weeks (*B* = 0.53; *p* > 0.05), but the differences were not statistically significant. The scores for self-efficacy of the MIG were higher than those of the CG at 8 weeks (*B* = 1.18; *p* > 0.05) and 12 weeks (*B* = 2.71; *p* < 0.05), but the differences were statistically significant only at 12 weeks (Table 4).

Further analysis was conducted on the subgroup of patients who were diagnosed with prostate cancer within two years, were not employed and had an annual household income of less than NT$500,000 (MIG = 17, CG = 15). The MIG’s social support (*B* = 0.77, *B* = 0.71; all *p* > 0.05) and self-efficacy (*B* = 0.71, *B* = 2.00; all *p* > 0.05) scores in both weeks 8 and 12 were higher than their respective pre-test scores. On the other hand, the CG’s social support (*B* = − 0.73, *B* = − 0.26; all* p* > 0.05) and self-efficacy (*B* = − 0.53, *B* = − 0.88; all *p* > 0.05) scores in both weeks 8 and 12 were lower than their respective pre-test levels; however, none of these changes reached statistical significance. In terms of social support and self-efficacy, the MIG scored higher than the CG at 8 (*B* = 1.50, *B* = 1.24; all *p* > 0.05) and 12 weeks (*B* = 0.78, *B* = 2.97; all *p* > 0.05); however, the differences were not statistically significant (Table 5).

### Satisfaction with the program

The patients in the MIG were satisfied with the multimedia-based HTIP and agreed that it was helpful in learning self-care. The feedback provided by patients included the following: “Overall satisfaction with the program” (80.0 ± 10.7); “The program is helpful to me” (78.7 ± 11.2); “The program tells me how to take care of myself” (80.0 ± 13.5); “The program increases my confidence in taking care of myself” (77.9 ± 12.7); and, “The program is suitable for future patients” (78.2 ± 14.9).

## Discussion

Based on the literature, social cognitive theory^32,33^, and the social cognitive transition model of adjustment^34^, a multimedia-based HTIP, provided once a week for 6 weeks, was developed. We posited that promoting individual cognitive, social environmental, and supportive behavioral factors through a multimedia-based HTIP once a week for 6 weeks would contribute to an improvement of patients’ social support and self-efficacy, and further modify their behavior toward a positive change, resulting in more positive thinking and better QOL. Previous studies have provided computer- and Internet-based education programs to patients with localized prostate cancer in Western countries^14,15,25,26,28^. However, the present study was conducted in Taiwan. The low recruitment rate (only two participants from each center per month) may have been due to the COVID-19 epidemic and its prevention regulations as well as short-staffed conditions at the hospital. Nevertheless, the recruitment rate was higher than 0.92 participants (median) from each center per month in 151 individually randomized controlled trials of a systematic review study^54^. The retention rate in the present study was 96.3%. The patients in the MIG felt that the program helped them to learn and increased their confidence in self-care. They also felt that the program could be provided to patients with prostate cancer who are undergoing hormone therapy. Moreover, none of the patients indicated that the program caused them harm. Therefore, this program is considered acceptable and feasible^56,57^. Finally, because those over 70 years old, as compared with younger individuals, may be less familiar with the instant messaging application on their smartphones, they may require more guidance and practice.

The results of this study showed that, compared with patients in the CG, patients in the MIG improved slightly in terms of positive thinking and prostate cancer-specific QOL at 8 weeks and 12 weeks after pre-test as well as general QOL at 8 weeks after pre-test; however, this improvement was not statistically significant. A previous study on patients with prostate cancer who exhibited the symptom of hot flushes after receiving hormone therapy and who were provided group-based cognitive behavioral intervention once a week for 4 weeks presented similar findings. The results showed that the intervention did not significantly improve the depression, anxiety, or overall QOL of the patients^16^. Previous research, however, has shown that a 10-week technology-assisted psychosocial intervention can mitigate depression in patients with advanced prostate cancer and improve their general QOL after 6 months of intervention^58^. The present study, however, providing the 6-week multimedia-based HTIP once a week, followed up with patients for only 12 weeks after completing the pre-test questionnaire, and the sample size was limited. Further studies with longer periods of intervention, long-term follow-up and a larger sample size, should focus on patients who have received or intend to undergo hormone therapy to determine the efficacy of the multimedia-based HTIP.

Social support is an important factor in improving patients’ self-efficacy and positive transition^32–34^. Furthermore, support from their intimate partners, families, friends, and relatives is also important when patients with prostate cancer are adapting to the impact of the disease and treatments. Unlike previous studies^29,58^, this study included only prostate cancer patients with their partner or significant other who lived and learned with the patient. Thus, this population has better social support than do prostate cancer patients who live alone or are single, which may be a potential reason that, although the patients in the MIG were satisfied with the intervention, the effect of the intervention was not significant. Future research may consider including patients who live alone, are single, or have weak support systems to understand the feasibility and effectiveness of intervention.

The results of this study showed that the self-efficacy of patients in the MIG and CG was similar at 8 weeks and 12 weeks after pre-test. It decreased and was lower in the MIG than in the CG at 8 weeks, however, and then slightly increased and was higher than that in the CG at 12 weeks. The results are similar to those of previous studies on patients with localized prostate cancer who were receiving surgery or radiation^15,26,28^. The results of a past study on prostate cancer patients who were undergoing hormone therapy for an average of 89 days (range = 0–730 days) in Canada, which adopted a single group pre-test and post-test study design, however, showed that patients’ self-efficacy in managing side effects could be significantly improved to an average of 81 days after receiving an education program comprised of a 1.5-h class and reading a book about hormone therapy^29^.

In addition, accumulated achievements based on a successful experience is one means to improve self-efficacy^32^. The actual successful experience of patients, however, may have been limited in this study due to the short follow-up time. A long-term follow-up intervention study is needed to clarify the efficacy of the program. Moreover, patients in different stages of their disease may face different disease conditions and have different information needs. The participants of this study were patients who were living with prostate cancer between 1 and 228 months who underwent hormone therapy. These differences were not taken into account in this study and could have contributed to the limited significant results.

Within two to three years after patients with prostate cancer underwent primary hormone therapy, the disease may progress to androgen-independent prostate cancer, which compels healthcare providers to adjust the treatment formula^3^. By focusing on patients with prostate cancer diagnosed within two years, this study uses a subgroup analysis to understand the preliminary outcomes of a multimedia-based HTIP. The results showed that, compared with the CG, the MIG tended to have better general QOL, prostate cancer-specific QOL, social support, and self-efficacy at 8 and 12 weeks after pre-test. Compared with the pre-test, the FACT-P mean scores of patients in the MIG increased by 4.25–4.50 points at 8 and 12 weeks after pre-test, and the mean scores were 4.57–4.76 points greater than those of the CG. Nevertheless, the difference was not clinically meaningful^45,46^. Moreover, the social support and self-efficacy of patients who received a multimedia-based HTIP in the MIG were slightly better than those in the CG at 8 and 12 weeks after pre-test. This implies that future randomized control studies could assess the efficacy of multimedia-based HTIP on patients with prostate cancer within two years.

In this study, disparities in pre-test basic attributes between MIG and CG included differences in the timing of diagnosis, annual household income, and employment status. Previous research has documented that economic status is a factor that affects individuals’ positive thinking and QOL^10,19^. We therefore conducted a subgroup analysis focusing on prostate cancer patients diagnosed within two years, who were not employed and had an annual household income of less than NT$500,000. In terms of primary and secondary outcomes, MIG consistently had better scores than the CG at both the 8 and 12 weeks after the pre-test. However, these differences did not reach statistical significance. Notably, MIG outperformed CG most strongly in general QOL (with differences ranging from 3.87 to 5.74 points) and prostate cancer-specific QOL (with differences ranging from 7.88 to 8.08 points). On the other hand, when comparing the post-test scores with the pre-test scores, MIG showed the greatest improvement in general QOL (an increase of 1.30–2.59 points) and prostate cancer-specific QOL (an increase of 3.89–4.65 points) at the 8 and 12 weeks after the pre-test. However, these improvements were not statistically significant or clinically meaningful^45,46^. This finding may be related to the lower QOL reported by patients within the tested subgroup (MIG = 76.88 for FACT-G and 112.76 for FACT-P) when compared with all participants (MIG = 79.86 for FACT-G and 116.39 for FACT-P) at the pre-test. Consequently, these patients had a greater potential for improvement. Because the present study constituted a pilot study with a small sample size, these findings may have been spurious. Further studies are needed to clarify the efficacy of multimedia-based HTIP on this patient subgroup.

Interestingly, this study found that MIG exhibited improved scores at the 8 weeks following the pre-test but experienced a slight decline at the 12 weeks across multiple variables, including positive thinking and QOL (general QOL, prostate cancer-specific QOL, and hormone symptoms and distress). In contrast, CG demonstrated slight improvements in these QOL scores at both the 8 and 12 weeks after the pre-test. This phenomenon may be attributed to the longer time since diagnosis for CG. Over time, patients tend to adapt to the changes brought about by the disease and develop coping strategies^18^. In the subsequent subgroup analysis, it was observed that MIG scores in positive thinking and QOL maintained similar trends, while CG QOL scores worsened at the 8 weeks after the pre-test, with some variables showing slight improvement at the 12 weeks. When accounting for the factor of time since diagnosis, MIG experienced a slight drop in scores at the 12 weeks. This decline may be associated with the challenge of sustaining the intervention efficacy over an extended period. Future research is necessary to gain a better understanding of the efficacy of multimedia-based HTIP for patients undergoing hormone treatment.

## Limitations

Prostate cancer patients with mental illness, who were incapable of taking care of themselves, who did not have a smartphone, or who were single and living alone were excluded from this study. These exclusions may limit the efficacy and the inferences of the preliminary results. Patients with prostate cancer who were in different disease stages, annual household income, and occupational status and underwent hormone therapy were included in this study but may have had differing needs for information. Moreover, this is a pilot study, and the sample size was small. Large-sample and multi-institutional randomized trials with long-term follow-up that include patients with high homogeneity in early-stage disease and basic information are warranted to determine the efficacy of the multimedia-based HTIP. The CG in the present study received routine care, which posed challenges in achieving a truly blinded design. Future research could explore the possibility of implementing an attention control group to enhance internal validity^59^. To enhance research feasibility, the present study aligned with the timing of the patients’ monthly clinic visits for hormonal drug injections. Consequently, post-test 1 occurred 8 weeks after the pre-test, following six weeks of intervention. Nevertheless, this may not provide a valid assessment of immediate post-intervention efficacy. Future research should aim to address this limitation. In addition, no data for cohabitants were collected in this study. Therefore, it was not possible to examine their views regarding the program. Future research could study the efficacy of the intervention program as assessed by various individuals, including patients and partners, using a mixed methods research design. Furthermore, this study did not explicitly restrict the control group from engaging in self-study methods that could potentially benefit their physical and mental health. This lack of differentiation between the MIG and CG may have contributed to the absence of significant differences in our findings. We recommend that future research should collect data on participant self-learning content and experiences and explore the potential influence of these factors on research outcomes.

## Conclusion

The use of a multimedia-based hormone therapy information program tended to improve positive thinking, prostate cancer-specific QOL, and social support in patients with prostate cancer who underwent hormone therapy, but the preliminary results did not reach statistical significance. Nevertheless, this study achieved a high retention rate, and the patients in the MIG were satisfied with the program and felt that the program was helpful in terms of learning self-care. The program may thus be feasible and acceptable for patients with prostate cancer undergoing hormone therapy. Randomized control studies with a larger sample size and long-term follow-up are required to verify the effectiveness of the program. Moreover, a multimedia-based HTIP provided for patients with prostate cancer who underwent hormone therapy could be provided as early as possible after receiving treatment and even prior to treatment.

## Data Availability

The data used in this study are stored and managed by the corresponding author, to whom readers can direct any questions. The data are not publicly available due to the consideration of ethics, the researchers shall maintain the privacy of the participants, and research data should be used only for academic.

## Abbreviations

BMI: Body mass index
CG: Control group
GEE: Generalized estimating equation
HTIP: Hormone therapy information program
FACT-G: Functional assessment of cancer therapy (FACT)-general
FACT-P: Functional assessment of cancer therapy (FACT)-prostate
MIG: Multimedia information group
PSA: Prostate-specific antigen
QOL: Quality of life
